# Diffusion Maps Clustering for Magnetic Resonance Q-Ball Imaging Segmentation

**DOI:** 10.1155/2008/526906

**Published:** 2008-02-10

**Authors:** Demian Wassermann, Maxime Descoteaux, Rachid Deriche

**Affiliations:** ^1^Odyssee Project Team INRIA/ENPC/ENS INRIA, Sophia-Antipolis, 2004 Route des Lucioles, 06902 Sophia Antipolis, France; ^2^Computer Science Department, FCEyN, UBA, Pabellón 1, Ciudad Universitaria, C1428EGA Buenos Aires, Argentina

## Abstract

White matter fiber clustering aims to get insight about anatomical structures in order to generate atlases, perform clear visualizations, and compute statistics across subjects, all important and current neuroimaging problems. In this work, we present a diffusion maps clustering method applied to diffusion MRI in order to segment complex white matter fiber bundles. It is well known that diffusion tensor imaging (DTI) is restricted in complex fiber regions with crossings and this is why recent high-angular resolution diffusion imaging (HARDI) such as Q-Ball imaging (QBI) has been introduced to overcome these limitations. QBI reconstructs the diffusion orientation distribution function (ODF), a spherical function that has its maxima agreeing with the underlying fiber populations. In this paper, we use a spherical harmonic ODF representation as input to the diffusion maps clustering method. We first show the advantage of using diffusion maps clustering over classical methods such as N-Cuts and Laplacian eigenmaps. In particular, our ODF diffusion maps requires a smaller number of hypothesis from the input data, reduces the number of artifacts in the segmentation, and automatically exhibits the number of clusters segmenting the Q-Ball image by using an adaptive scale-space parameter. We also show that our ODF diffusion maps clustering can reproduce published results using the diffusion tensor (DT) clustering with N-Cuts on simple synthetic images without crossings. On more complex data with crossings, we show that our ODF-based method succeeds to separate fiber bundles and crossing regions whereas the DT-based methods generate artifacts and exhibit wrong number of clusters. Finally, we show results on a real-brain dataset where we segment well-known fiber bundles.

## 1. INTRODUCTION

Recent work shows that diffusion magnetic resonance imaging (dMRI) can help recovering complex white matter fiber bundles. However this is still an open problem due to the structural complexity of the fiber bundles, which can have crossing configurations. Diffusion tensor imaging (DTI) [1] is restricted in these conditions due to the hypothesis that the diffusion within a voxel follows a Gaussian distribution, a model that cannot model intravoxel crossings. Q-ball imaging (QBI) [2], a recent high-angular resolution diffusion imaging (HARDI) technique, overcomes this limitation by reconstructing a diffusion orientation distribution function (ODF), a spherical function that has its maxima agreeing with the underlying fiber populations. The ODF reconstruction from QBI is attractive because it is model-free and has been recently shown possible with a regularized and analytical solution [3], which produces a robust and very fast ODF reconstruction. In fact, the ODF estimation is, in practice, as fast as a standard least-square diffusion tensor (DT) estimation.

Efficient segmentation of fiber tracts in dMRI images is an important problem in neuroimaging problem because it has many potential applications. For example, it could potentially provide important information on diseases that affect fiber tracts. Alteration of the fiber tracts may provide new biomarkers in white matter pathologies and segmentation of these tracts can also improve our understanding of the functional role these tracts have and the cognitive consequences of their disruption.

The goal of this work is to provide a segmentation method that can separate the main white matter fiber bundles in the brain. We propose a new method that can segment fiber bundles and deal with fiber crossings while also requiring a minimum number of hypothesis from the data and a small number of algorithmic parameters. Spectral embedding and clustering methods have recently proved to be effective in image segmentation [4, 5]. However, classical approaches require restrictive hypotheses that are difficult to meet in real applications. For instance, N-Cuts [4] and Laplacian eigenmaps [6] require data within each cluster to be uniformly sampled, which produces artifacts when this hypothesis is not met. Moreover, classical approaches for image segmentation also assume that the scale within each cluster is the same using a single-scale parameter for the whole dataset. In order to overcome these limitations, we propose to use diffusion maps [7] as spectral embedding method. This method looses the dependence on the sampling of the elements to cluster. Moreover, we propose to use an adaptive scale-space parameter in order to deal with space-scale differences across different clusters. Finally, we propose two approaches to automatically determine the number of clusters by analyzing the spectra of the image embedding.

Another contribution of this paper is to show that the Q-ball ODF clustering using diffusion maps can reproduce the DT clustering using N-Cuts on simple synthetic images without crossings. On more complex data with crossings, we show that our method succeeds to separate fiber bundles and crossing regions on synthetic data, where the DT-based methods generate artifacts and exhibit wrong number of clusters. Finally, we successfully segment the fiber bundles in a real-human brain dataset in different regions with fibers crossing.

## 2. METHODS

The main goal of this work is to produce a segmentation algorithm able to segment white matter fiber bundles from dMRI data. In order to represent intravoxel crossings with the ODF, we need at least 15 real coefficients when a spherical harmonic basis is used [3, 8, 9]. This leads to 3D images with a high dimensional element at each voxel. This high dimensionality makes previous diffusion imaging segmentation approaches based on level set methods such as [10–12] computationally expensive. Moreover, these methods require an initialization step. In order to perform the segmentation in an initialization-free manner and with a lower-dimensionality image, we use spectral clustering methods [4, 5] which perform dimensionality reduction before performing the segmentation and do not need initialization. The segmentation is then performed on the statistics within each cluster and the fiber crossings can be identified.

In this section, we present the three main parts of our algorithm. First, the estimation of the Q-ball diffusion ODF and its compact representation using spherical harmonics. Second, the metric used to measure distances between Q-ball ODFs. Last, the diffusion aps spectral clustering technique used to segment the ODF image into the background and the different fiber bundles.

### 2.1. ODF estimation from QBI

**Figure 1:**
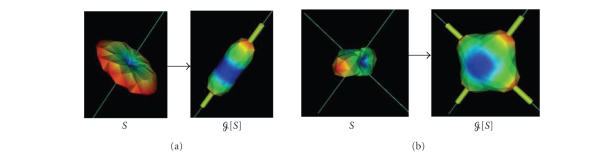
Funk-Radon transform G illustrated for the input diffusion attenuation signal S (b=1000 s/mm^2^) with 1 fiber (left) and two orthogonal fibers (right). The thin lines are the true underlying fiber directions and the thicker tubes are the detected maxima. One must imagine these functions as living on the surface of the sphere. Here, for visualization purposes, the radius of the respective spheres are scaled by the corresponding value on the surface. Blue-to-red colors represent low-to-high spherical values.

QBI [2] reconstructs the diffusion ODF directly from the HARDI measurements on a single sphere by the Funk-Radon transform (FRT). In practice, the FRT value at a given spherical point is the great circle integral of the signal on the sphere defined by the plane through the origin with normal vector. The FRT is qualitatively illustrated in Figure 1. The ODF is intuitive because it has its maximum(a) aligned with the underlying population of fiber(s). However, computing statistics on a large number of discrete ODF values on the sphere is computationally heavy and infeasible to integrate into a segmentation algorithm of the whole brain. A more compact representation of the ODF is thus needed. In [3, 8, 9, 13] a simple analytic spherical harmonic (SH) reconstruction of the ODF is proposed. For completeness of the article, we now review and develop the main parts of our regularized analytical ODF reconstruction solution. The idea is to first estimate HARDI signal on the sphere with a regularized spherical harmonics approximation and then do a simple linear transformation of the harmonics to obtain the desired regularized ODF.

**Spherical harmonic (SH) estimation of the HARDI signal**

The SH, normally indicated by $Y_{\ell }^{m}$ (ℓ denotes the order and m the phase factor), are a basis for complex functions on the unit sphere. Explicitly, they are given as follows:

(1) $$Y_{\ell }^{m}(\theta ,\phi )=\sqrt{\frac{2\ell +1}{4\pi }\frac{(\ell -m)!}{(\ell +m)!}}P_{\ell }^{m}(\cos\theta )e^{\text{i}\text{m}\phi },$$

where (θ,ϕ) obey physics convention (θ∈[0,π],ϕ∈[0,2π]) and $P_{\ell }^{m}$ is an associated Legendre polynomial. For k=0,2,4,…,ℓ and m=−k,…,0,…,k, we define the new index $j:=j(k,m)=(k^{2}+k+2)/2+m$ and define our modified basis Y with elements $Y_{j}$ such that

(2) $$Y_{j}=\{\begin{array}{ll} \sqrt{2}\cdot \text{R}\text{e}(Y_{k}^{m}) & \text{i}\text{f}\;-k\leq m<0,\\ Y_{k}^{0} & \text{i}\text{f}\;m=0,\\ \sqrt{2}\cdot \text{I}\text{m}\text{g}(Y_{k}^{m}) & \text{i}\text{f}\;0<m\leq k, \end{array}$$

where Re($Y_{\ell }^{m}$) and Img($Y_{\ell }^{m}$) represent the real and imaginary parts of $Y_{\ell }^{m}$, respectively. The basis is designed to be symmetric, real, and orthonormal. Symmetry is ensured by choosing only even order SH and the ratios in front of each term also ensure that the modified basis is real and orthonormal with respect to the inner product $\langle f,g\rangle =\int_{\Omega }f^{*}gd\Omega$, where Ω denotes integration over the unit sphere and $f^{*}$ is the complex conjugate of f for f and g complex functions on the sphere. We thus approximate the signal at each of the N gradient directions i as

(3) $$S(\theta_{i},\phi_{i})=\overset{R}{\underset{j=1}{\sum }}c_{j}Y_{j}(\theta_{i},\phi_{i}),$$

where R=(ℓ+1)(ℓ+2)/2 is the number of terms in the modified SH basis Y of order ℓ. Letting S be the N × 1 vector representing the input signal for every encoding gradient direction, C the R × 1 vector of SH coefficients $c_{j}$, and B is the N × R matrix constructed with the discrete modified SH basis

(4) $$B=\left(\begin{array}{cccc} Y_{1}(\theta_{1},\phi_{1}) & Y_{2}(\theta_{1},\phi_{1}) & \cdots & Y_{R}(\theta_{1},\phi_{1})\\ \vdots & \vdots & \ddots & \vdots \\ Y_{1}(\theta_{N},\phi_{N}) & Y_{2}(\theta_{N},\phi_{N}) & \cdots & Y_{R}(\theta_{N},\phi_{N}) \end{array}\right).$$

We can write the set of equations as an overdetermined linear system S=BC. We want to solve for the SH series coefficients $c_{j}$, where $c_{j}=\int_{\Omega }S(\theta ,\phi )Y_{j}(\theta ,\phi )d\Omega$.

At this point, instead of simply evaluating the integrals directly as done in [14] or performing a straightforward least-squared minimization as in [15, 16], we add local regularization directly into our fitting procedure. This is to be able to use a high-order estimation without overmodeling the small perturbations due to noise in the input diffusion MRI signal. We thus define a measure, E, of the deviation from smoothness of a function f defined on the unit sphere as $E(f)=\int_{\Omega }{\left({▵}_{b}f\right)}^{2}d\Omega$, where ${▵}_{b}$ is the Laplace-Beltrami operator. Using the orthonormality of the modified SH basis, where we have $\int_{\Omega }Y_{i}(\theta ,\phi )Y_{j}(\theta ,\phi )d\Omega =\delta_{ij}$, the above functional E can be rewritten straightforwardly [3, 13] as

(5) $$\begin{array}{ll} E(f) & {=\int }_{\Omega }{▵}_{b}\left(\underset{p}{\sum }c_{p}Y_{p}\right){▵}_{b}\left(\underset{q}{\sum }c_{q}Y_{q}\right)d\Omega \\ & =\overset{R}{\underset{j=1}{\sum }}c_{j}^{2}\ell {\left(j\right)}^{2}{\left(\ell (j)+1\right)}^{2}=C^{\text{T}}LC, \end{array}$$

where L is simply the R×R matrix with entries $\ell {\left(j\right)}^{2}{\left(\ell (j)+1\right)}^{2}$ along the diagonal (ℓ(j) is the order associated with the jth coefficient, that is, for j=1,2,3,4,5,6,7,…ℓ(j)=0,2,2,2,2,2,4,…). We thus obtain a closed-form expression for the regularization term. Therefore, the quantity we wish to minimize can be expressed in matrix form as

(6) $$M(C)=(S-BC{)}^{\text{T}}(S-BC)+\lambda C^{\text{T}}LC,$$

where λ is the weight on the regularization term. The coefficient vector minimizing this expression can then be determined just as in the standard least-squares fit (λ=0) from which we obtain the generalized expression for the desired spherical harmonic series coefficient vector

(7) $$C=(B^{\text{T}}B+\lambda L{)}^{-1}B^{\text{T}}S.$$

From this SH coefficient vector we can recover the signal on the Q-ball for any (θ,ϕ) as $S(\theta ,\phi )=\sum_{j=1}^{R}c_{j}Y_{j}(\theta ,\phi ).$ Intuitively, this approach penalizes an approximation function for having higher-order terms in its modified SH series. This eliminates most of the higher-order terms due to noise while leaving those that are necessary to describe the underlying function. However, obtaining this balance depends on choosing a good value for the parameter λ. We use the *L-curve* numerical method [17] and experimental simulations to determine a good smoothing parameter [3, 13, 18]. Here, λ=0.006 is used as in [3, 13, 18].

**Analytical ODF estimation**

The true diffusion orientation distribution function (ODF) in a unit direction u, Ψ(u), is given by the radial projection of the probability distribution function (PDF) of the diffusing water molecule. Tuch [2] showed that this diffusion ODF could be estimated directly from the raw HARDI signal S on a single sphere of Q-space by the Funk-Radon transform (FRT) (Figure 1). In [3, 13], we showed how this FRT can be evaluated analytically with an elegant corollary to the Funk-Hecke theorem [19]. The final ODF reconstruction on the sphere then becomes a simple linear transformation of the SH coefficients $c_{j}$ describing the input HARDI signal S,

(8) $$\Psi (\theta ,\phi )=\sum_{j=1}^{R}\underset{f_{j}}{\underset{︸}{2\pi P_{\ell (j)}(0)c_{j}}}Y_{j}(\theta ,\phi ),$$

where $f_{j}$ are the SH coefficients describing the ODF Ψ and $P_{\ell (j)}(0)=(-{1)}^{\ell /2}(1\cdot 3\cdot 5\cdots (\ell (j)-1)/2\cdot 4\cdot 6\cdots \ell (j))$ because ℓ(j) is always even in our modified SH basis. We see that the SHs are eigenfunctions of the Funk-Radon transform with eigenvalues depending only on the order ℓ of the SH series.

Hence, by using an SH estimation of the HARDI signal, we have showed that the QBI can be solved analytically. This was also showed in [8, 9]. An important contribution in favor of our approach is that this solution can be obtained while imposing a well-defined regularization criterion. The accuracy of the modified SH series approximation with the Laplace-Beltrami smoothing was established in [18] and our regularized ODF solution was also shown to have better fiber detection properties and shown to be more robust to noise than similar solutions [8, 9].

### 2.2. Distances between ODFs

Once the ODF are computed, we want to capture similarities and dissimilarities between two ODFs, that is, two spherical functions $\Psi ,\Psi^{\prime }\in S^{2}$ that can be represented by real-SH vectors of length R, $f=\{f_{1},\ldots ,f_{R}\}$ and $f^{\prime }=\{f_{1}^{\prime },\ldots ,f_{R}^{\prime }\}\in {\mathbb{R}}^{R}$, as shown in (8) in the previous section. Since the ODFs come from real physical diffusion measurements they are bounded and form an open subset of the space of real-valued ${ℒ}^{2}$ spherical functions with an inner product 〈,〉 defined as

(9) $$\begin{array}{ll} \langle \Psi ,\Psi^{\prime }\rangle & {=\int }_{\Omega }\Psi (\theta ,\phi )\cdot \Psi (\theta ,\phi {)}^{\prime }d\Omega \\ & {=\int }_{\Omega }\left(\overset{R}{\underset{i=1}{\sum }}f_{i}Y_{i}(\theta ,\phi )\overset{R}{\underset{j=1}{\sum }}f_{j}^{\prime }Y_{j}(\theta ,\phi )\right)d\Omega . \end{array}$$

Again, because of the orthonormality of the spherical harmonic basis, the cross-terms cancel and the expression is simply

(10) $$\langle \Psi ,\Psi^{\prime }\rangle =\overset{R}{\underset{j=1}{\sum }}f_{j}\cdot f_{j}^{\prime }.$$

Therefore, the induced ${ℒ}^{2}$ norm $\left\|\Psi \right\|=\sqrt{\langle \Psi ,\Psi^{\prime }\rangle }$ giving us the distance metric between two ODFs is

(11) $$\left\|\Psi -\Psi^{\prime }\right\|=\sqrt{\overset{R}{\underset{j=1}{\sum }}{\left(f_{j}-f_{j}^{\prime }\right)}^{2}}.$$

The Euclidean distance was also used successfully for ODF segmentation in [12] and for DTI segmentation in [11] even though more appropriate metrics exist such as the J-divergence [11, 20] and Riemannian geodesic distances [11]. Similarly, one can think of choosing another metric to compare ODFs. For instance, since the ODF can be viewed as a probability distribution function (PDF) of fiber orientations, one can use the Kullback-Leibler distance between two PDFs, as done in [2]. However, in that case the problem quickly blows up computationally because one needs to use all N discrete HARDI data on the sphere instead of the R SH coefficients (R≪N).

### 2.3. Diffusion maps-based clustering

We now want to segment white matter fiber bundles in a Q-ball image. One of the open questions in Q-ball image analysis and clustering is that which metric should be used to compare Q-ball ODFs. Here, we describe a clustering algorithm that infers an embedding and a metric to compare ODF images. We derive an affinity measure incorporating the Euclidean distance and the spatial location distance between ODFs. This affinity measure then used in a spectral embedding framework. As mentioned in [7], the Euclidean distance within this embedding actually represents an *intrinsic metric of the data*, which can be used to perform statistics in the embedded space and can thus be used to segment Q-ball ODF images into white matter fiber bundles.

**Spectral embedding and clustering**

In recent years, spectral manifold learning and clustering techniques [4, 6, 21–23] have become one of the most popular modern clustering family of methods. They are simple to implement, they can be solved efficiently by standard linear algebra software, and they very often outperform traditional manifold learning and clustering algorithms such as the classical principal component analysis (PCA) [24] and k-means [25] algorithms. Moreover, due to the dimensionality reduction properties, they are especially well suited to work with high-dimensional data. These techniques have been recently used to cluster various types of images [4, 5] and white matter fiber tracts [26]. In our case, we perform the spectral clustering for two different types of elements: the DT and the ODF. In the DT case, the element is represented by a 6-dimensional vector corresponding to the upper (or lower) triangular part of the DT 3×3 symmetric matrix. In the ODF case, the element is represented by the 15-dimensional vector corresponding to the 4th-order spherical harmonic ODF estimation.

Spectral clustering reduces the clustering problem to a graph partitioning problem. Each element to be clustered is represented as a node in a graph and the edges joining the vertex are a measure of affinity between the elements. This affinity measure lies between 0 and 1, 0 being the less affine case. A spectral decomposition of this graph is taken by calculating the eigenvalue decomposition (EVD) of the graph Laplacian [27]. Then a low-dimensional Euclidean manifold embedding is inferred from this decomposition. Finally, the clustering is performed in the inferred Euclidean manifold.

All the above techniques rely on three hypotheses.

1. Preservation of the distance relationship: after a distance is defined between elements, the learned manifold should preserve the distance relation.
2. Uniform sampling of the elements: the density of the extracted elements changes if and only if these elements belong to anatomically different bundles.
3. Convexity of the elements: if two elements are in the dataset, almost all of the intermediate tracts obtained by the interpolation that can be inferred from the metric used to build the affinity matrix are in the dataset.

It is not easy to guarantee that the data to be embedded and clustered will adhere to these hypotheses. Donoho and Grimes, in [13], analyze when a spectral embedding algorithm is able to recover the true parameterization of a set of images. As medical images represent the discretization of a continuous space, hypotheses 1 and 3 are plausible. However, there is no indication that within a fiber bundle the distribution of the elements (DT or ODF) are uniformly sampled. Moreover, in [29]it is shown that different sampling frequencies within one cluster leads the N-Cuts and Laplacian eigenmaps methods to subdivide the cluster in several parts. In order to overcome this limitation and to be resilient to sampling frequency differences within a cluster, we use the diffusion maps [7] spectral embedding technique. We now describe the three steps involved in the diffusion maps algorithm in turn.

***Step 1* (Computing the affinity matrix).**

Letting X represent the set of all ODF elements to cluster, the main idea is to look for a representation between the elements of X that is more representative than ${\mathbb{R}}^{R}$ (recall that ODFs are $\in {\mathbb{R}}^{R}$) and reduces the dimensionality of the problem. With keeping this in mind, a fairly good way of representing any set of elements with an affinity function $a:X\times X\to {\mathbb{R}}_{>0}$, is a weighted graph, G(X,E,w(·)), where the weight of the edge between two vertices represents the affinity of the elements connected by this edge. More formally, for an edge,^1^ $e=(f_{i},f_{j})\in E$, the weight of the edge is $w(e)=a(f_{i},f_{j})$. Hence, each element of the adjacency matrix of G or conversely the affinity matrix of (X,a(·)) is

(12) $$A_{ij}=a(f_{i},f_{j}).$$

Taking this in account, the weighted graph G(X,E,w(·)) can be also noted as G(X,A).

Usually, a distance function d(·) instead of an affinity function is given. The distances can be easily converted into affinities by applying a kernel to the distance function

(13) $$a(f_{i},f_{j})=e^{-(d{\left(f_{i},f_{j}\right)}^{2}/\sigma_{ij}^{2})},$$

where σ is an adaptive scale-space parameter that may depend on the elements $f_{i}$ and $f_{j}$. In this work, the adaptive scale-space parameter is taken following [30]. A “neighbor-number” k is given as parameter to the algorithm and then $\sigma_{ij}^{2}=d(f_{i},f_{i_{k}})d(f_{j},f_{j_{k}})$, where $f_{i_{k}}$ is the kth closest neighbor according to the distance function d(·,·) of element $f_{i}$. This choice of a scaling parameter for each point allows self-tuning of the point-to-point distances according to the local statistics of the neighborhoods surrounding points i and j.

As in image segmentation, the spatial position of each element is important, the spatial dependency should be incorporated into the affinity matrix. Following [5, 31], we use Markovian relaxation to incorporate this information. In order to represent the affinity of all the elements that can be reached within one spatial step, the affinity matrix is modified in the following way:

(14) $$A_{1}=\{\begin{array}{cc} A_{ij} & \text{if}\;\;∥\text{c}\text{o}\text{o}\text{r}\text{d}\text{s}(f_{i})-\text{c}\text{o}\text{o}\text{r}\text{d}\text{s}(f_{j}){∥}_{2}\leq 1,\\ 0 & \text{in}\;\;\text{any other case}, \end{array}$$

where coords (f) are spatial coordinates of element f in the image

(15) $$P_{1}=\frac{1}{\underset{l}{\max}D{\left(A_{1}\right)}_{ll}}\{\begin{array}{ll} \underset{l}{\max}D{\left(A_{1}\right)}_{ll} & \\ -D{\left(A_{1}\right)}_{ii} & \text{if}\;\;i=j,\\ A_{1\;\;} & \text{in}\;\;\text{any other case}, \end{array}$$

where $D(A_{1})$ is a diagonal matrix with $D{\left(A_{1}\right)}_{ii}=\sum_{j}A_{1ij}$, usually called the row-sum or degree matrix of $A_{1}$.

Then, obtaining the affinities of elements that can be reached within s spatial steps is enough to elevate $P_{1}$ to the power of s, $P_{s}=(P_{1}{)}^{s}$ as stated in [31]. Moreover, s can be chosen to be the smallest positive integer which results in nonzero elements in the whole matrix in order to represent the weakest connected induced graph. The diagonal adjustment forces the inherent random walk to a uniform steady state, hence every part of the Markov field will be explored at the same speed. For the sake of clarity, $P_{s}$ will be referred to as affinity matrix A in the rest of the paper.

***Step 2* (Performing the embedding).**

The algorithm must embed the elements of X into an n-dimensional Euclidean space y(X). This is done by applying eigenvalue decomposition to the Laplacian of the affinity matrix. This embedding must be compliant with hypothesis 1. As in [6, 7, 27], this is done by performing the spectral decomposition of the graph Laplacian of the graph induced by A,

(16) $$\Delta =D(A)-A\in {\mathbb{R}}^{\left|X|\times |X\right|},$$

where |X| is number of elements to be clustered.

In order to overcome the necessity of hypothesis 2, we prenormalize the affinity matrix as done in [7]. This is done by normalizing the weight of each edge of the graph, $A_{ij}$, by the probability density of both elements relating through the edge,

(17) $${\left(A_{p}\right)}_{ij}=\frac{A_{ij}}{p(i)p(j)},$$

where p(·), the probability density function of the elements in X, is not known but can be approximated up to a multiplication factor by

(18) $$p(i)=\sum_{k}A_{ik}=\sum_{k}A_{ki}.$$

Due to the necessity of having a uniform behavior of the clustering algorithm without minding the scale of the affinity measure taken, a doubly stochastic matrix normalization is performed:

(19) $$A_{\text{ds}}=D{\left(A_{p}\right)}^{-1/2}A_{p}D{\left(A_{p}\right)}^{-1/2}\in {\mathbb{R}}^{\left|X|\times |X\right|}.$$

As $A_{\text{d}\text{s}}$ is a double stochastic symmetric matrix, the eigenvalue decomposition of (16) can be calculated by taking the singular value decomposition (SVD)

(20) $$VSV^{T}=A_{\text{ds}}\in {\mathbb{R}}^{\left|X|\times |X\right|}.$$

Finally, the Euclidean coordinates $y_{i}$ of an element $f_{i}\in X$ in the n-dimensional embedding manifold are

(21) $$y(f_{i})=y_{i}=\frac{1}{v_{i}^{0}}{\left(\lambda_{1}v_{i}^{1},\ldots ,\lambda_{n}v_{i}^{n}\right)}^{T},{\;f}_{i}\in X,$$

where

(22) $$V=\left(v^{0}\cdots v^{|X|-1}\right)\in {\mathbb{R}}^{\left|X|\times |X\right|}$$

is the eigenvector column matrix and the corresponding eigenvalues are, $1=\lambda_{0}\geq \lambda_{1}\geq \cdots \geq \lambda_{|X|-1}\geq 0$. The first eigenvector $v_{0}$ is not taken into account as a component in the embedding because it is constant and hence meaningless as shown in [6, 7, 27].

***Step 3* (Clustering).**

Once the embedding has been performed, several techniques have been proposed for the clustering step [4, 6, 32].

The first step in this process is to determine the number of clusters, this can be done in two ways. The first, as in [33], is choosing the number of clusters according to the “elbow”. This is present in the eigenvalue plot. For instance, if the slope of the eigenvalue plot changes noticeably at eigenvector $\lambda_{i}$, the number of clusters should be i+1. The second way is reordering the affinity matrix rows and columns following the second eigenvector as proved in [34], which shows the block structure of the matrix as squared blocks along the matrix diagonal. Then, the number of clusters is the number of blocks. Their commended number of dimensions for the embedding is the same as the number of clusters. Finally, the clustering is performed by running a k-means clustering algorithm on the space spanned by y(X). A formal justification for this approach can be found in [6, 32].

### 2.4. Q-ball data generation and acquisitions

**Synthetic data**

We generate synthetic HARDI data using the multitensor model which is simple and leads to an analytical expression of the ODF [2, 18]. For a given b-factor and noise level, we generate the diffusion-weighted signal

(23) $$S(u_{i})=\overset{n}{\underset{k=1}{\sum }}\frac{1}{n}\exp(-b{\;u}_{i}^{\text{T}}D_{k}(\theta )u_{i})+\text{n}\text{o}\text{i}\text{s}\text{e,}$$

where $u_{i}$ is the ith gradient direction on the sphere, n is the number of fibers, and 1/n is the volume fraction of each fiber. In practice, we use N=81 from a 3rd-order tessellation of the icosahedron, b=3000 s/mm^2^, and n=1 or 2. $D_{k}(\theta )$ is the diffusion tensor with standard eigenvalues [3,3,1.7] ×10^−2^ mm^2^/s oriented in direction θ, which agree with reported physiological values [35]. Finally, we add complex Gaussian noise with standard deviation of 1/35, producing a signal with signal-to-noise ratio of 35.

We generate three synthetic data example, two simple examples: one with a ring of sinusoidal-shaped fibers, one with fibers with different sizes and scales, and the other with complex crossing areas simulating the “U”-fibers (corticocortical fibers) that can occur in the brain. These synthetic datasets help understand the behavior of the different spectral clustering methods when confronted with simple and complex fiber geometries.

**Human brain data**

Diffusion-weighted data and high-resolution T1-weighted images were acquired on a whole-body 3 Tesla Magnetom Trio scanner (Siemens, Erlangen) equipped with an 8-channel head array coil [36]. The spin-echo echo-planar-imaging sequence, TE=100 ms, TR=12 s, 128 × 128 image matrix, FOV = 220 × 220 mm^2^, consists of 60 diffusion encoding gradients [37] with a b-value of 1000 s/mm^2^. Seven images without any diffusion weightings are placed at the beginning of the sequence and after each block of 10 diffusion-weighted images as anatomical reference for offline motion correction. The measurement of 72 slices with 1.7 mm^2^ thickness (no gap) covered the whole brain. Random variations in the data were reduced by averaging 3 acquisitions, resulting in an acquisition time of about 45 minutes. No cardiac gating was employed to limit the acquisition time. The issue of cardiac gating is discussed in [38]. Additionally, fat saturation was employed and we used 6/8 partial Fourier imaging, a Hanning window filtering, and parallel acquisition (generalized autocalibrating partially parallel acquisitions, reduction factor = 2) in the axial plane.

The brain is peeled from the T1-anatomy, which was aligned with the Talairach stereotactical coordinate system [39]. The 21 images without diffusion weightings distributed within the whole sequence were used to estimate motion correction parameters using rigid-body transformations [40], implemented in [41]. The motion correction for the 180 diffusion-weighted images was combined with a global registration to the T1 anatomy computed with the same method. The gradient direction for each volume was corrected using the rotation parameters. The registered images were interpolated to the new reference frame with an isotropic voxel resolution of 1.72 mm^2^ and the 3 corresponding acquisitions and gradient directions were averaged.

**Distance functions between elements to cluster**

In order to implement the diffusion maps spectral clustering method a distance function for each data type is chosen. This distance function is used to calculate the affinity matrix as expressed by (13). In the DT case, following [42], we use the Riemannian tensor distance. In the ODF case we use the distance shown in (11).

## 3. RESULTS

### 3.1.Synthetic data experiments

**Diffusion maps versus N-cuts**

**Figure 2:**
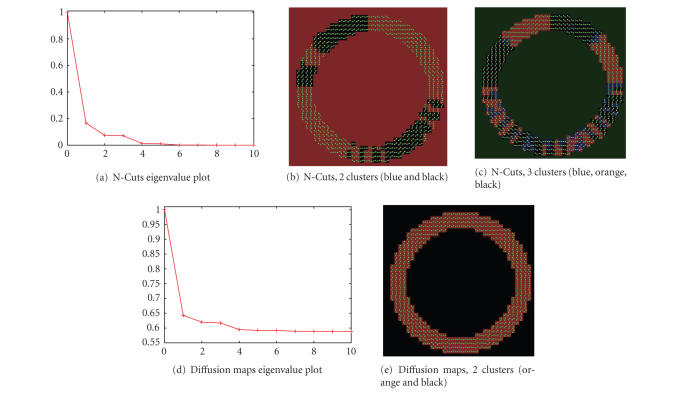
N-Cuts generates overclustering due to sampling frequency variation in ODF images. In both eigenvalue plots Figures 2(a) and 2(d), the slope between the line joining $\lambda_{0}$ and $\lambda_{1}$ and the line joining $\lambda_{1}$ and $\lambda_{2}$ changes drastically, expressing an elbow in $\lambda_{1}$, which indicates two clusters. The clustering results with Figures 2, 2(b), 3, 2(c), clusters are shown. Diffusion maps correctly finds two clusters, the object and the background, Figure 2(e). In the labeling, the ODFs are overlaid on the labels.

The first experiment shows the difference in performance between the diffusion maps and N-Cuts approach. The N-Cut algorithm does not perform the sampling-based normalization described by (17) and is thus sensitive to sampling frequency differences within the clusters. In order to show this sampling hypothesis problem, we used a ring fiber bundle with different sampling frequencies. Within the ring, the fibers have a sinusoidal shape and the frequency of the modulating sine function is 4 times bigger in the lower half of the ring. More formally, the fibers follow the angular function o(θ)=θ+(1/8)πsin⁡(μ·θ),0≤θ<2π, where μ=8 for the upper half of the ring and μ=32 for the lower half. Two clusters are expected, the ring and the background. The results of both clustering techniques are shown in Figure 2, where the background has been masked out. Figure 2(a) shows the plot of the first 10 eigenvalues for the N-Cuts method, shown in Figures 2(b) and 2(c), and the slope between the line joining $\lambda_{0}$ and $\lambda_{1}$ and the line joining $\lambda_{1}$ and $\lambda_{2}$ changes drastically. This elbow at $\lambda_{1}$ indicates that there are 2 clusters. Figure 2(d) shows the plot of the first 10 eigenvalues for the diffusion maps method whose clustering results are shown in Figure 2(e). The N-Cuts exhibits frequency-dependent clustering artifacts while the diffusion maps method clearly shows two clusters. In the diffusion maps, the clustering has correctly segmented the background and the ring.

**ODF versus DT images**

**Figure 3:**
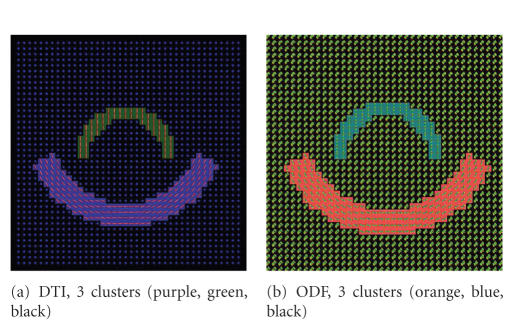
Synthetic image without fiber crossings. The results for the DT and ODF images are equivalent. The colors behind the DTs and ODFs indicate the clusters.

In Figure 3, a single fiber scenario with no fiber crossing is shown. The DT-based and ODF-based image clustering produce the same results. Hence, ODF clustering reproduces DT-based results on a simple fiber population example.

**Figure 4:**
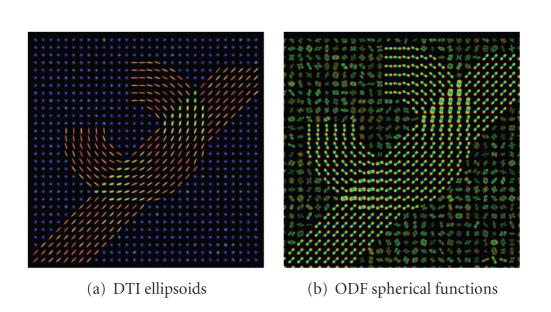
Synthetic DT and ODF images. The expected number is four, one for each fiber, one for the crossing between the two fibers and one for the background.

**Figure 5:**
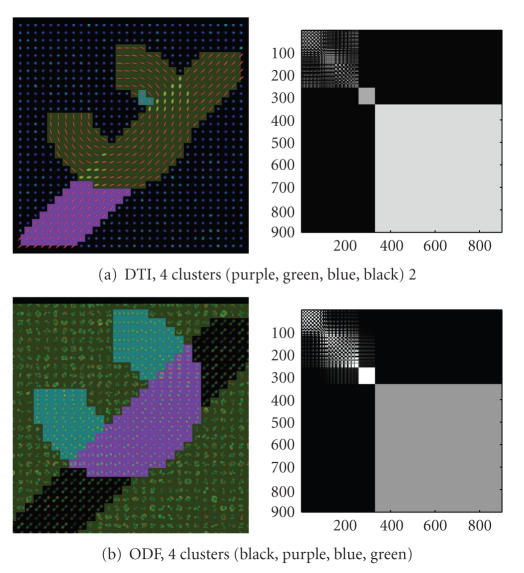
Clustering results in ODF and DT images, only ODF show the correct clustering. In both cases the clustering result and the reordered affinity matrix are shown.

Finally, Figure 4 shows a fiber crossing scenario with two overlapping fiber bundles that have different geometries. Segmentation was performed over the DT and the ODF image shown in Figure 5. Note that the cluster number is correctly estimated only in the ODF image. Moreover, the ODF N-Cuts segmentation exhibits artifacts not present in the ODF diffusion maps segmentation. The ODF diffusion maps effectively identify the two different fiber bundles as well as the fiber crossing areas.

### 3.2. Real data

The real-data experiment presented in this section shows the segmentation and labeling of a cropped axial and coronal slice. The cropped slices were chosen by an expert in regions of known fiber crossings where the DT model is normally limited. The ROIs show intersection of several fiber bundles. Hence, our segmentation algorithm is confronted with elements that have different orientation and different diffusion characteristics.

**Figure 6:**
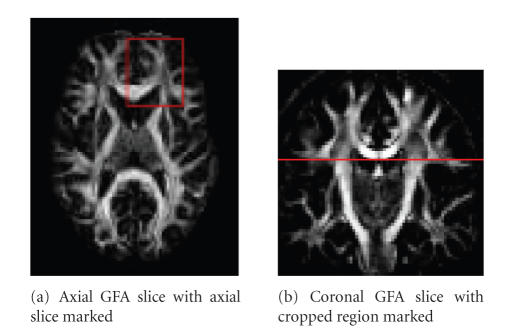
Generalized fractional anisotropy axial, Figure 6(a), and coronal, Figure 6(b) slices in the real dataset.

**Figure 7:**
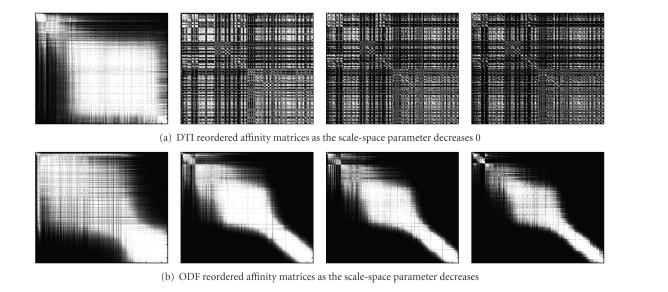
Plots of DTI and ODF affinity matrices of an axial cropped slice shown in Figure 6. The matrices are reordered according to the second (Fiedler) eigenvector. ThePlots of DTI affinity matrices are shown in decreasing order of σ, which takes the values 1/5, 1/10, 1/20, and 1/40 of the quantity of elements to cluster. In the DTI case, the decreasing on the scale parameter σ leads to a matrix with highly correlated elements that is very difficult to cluster. In the ODF case, the block structure is clear and is better suited to apply a clustering algorithm.

In order to show that ODF data segments the white matter fiber bundles better than the DT data in real cases, we analyze the evolution of the affinity matrix as the scale-space parameter changes in the axial cropped slice shown in Figure 6. Affinity matrices were computed with varying scale-space parameter between 1/5, 1/10, 1/20, and 1/40 of the quantity of elements (|X|) to cluster, respectively. In order to show the block structure of the affinity matrices, they were reordered using the second (Fiedler) biggest eigenvector [34]. It can be seen in Figure 7 that as the scale diminishes, the DT data shows a high correlation between all the elements of the slice. This makes clustering very difficult because the blocks are small and highly correlated. On the other hand, the ODF data shows a very clear block structure across all scales. This block structure shows a high correlation of the elements within each block and a low interblock correlation, giving a much better input to the clustering algorithm than the DT data.

**Figure 8:**
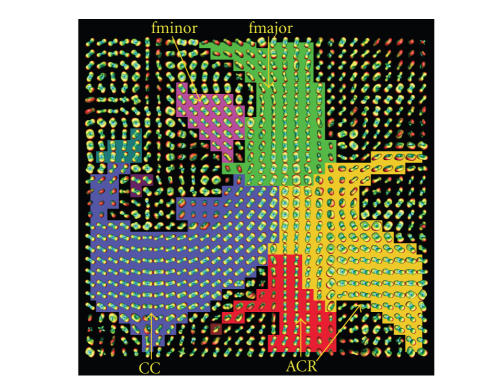
Our proposed algorithm is able to identify important white matter fiber bundles on an axial slice of a real dataset. The cropped axial slice shown in Figure 6(a) has been segmented. In the labeled ODF visualization, each color represents one of the clusters found. The white matter labels are CC: Corpus Callosum, ACR: Anterior Corona Radiata, fmajor: Forceps Major and fminor: Forceps Minor.

In Figure 6, the location of the cropped axial slice is shown in the axial slice, Figure 6(a), and coronal slice, Figure 6(b). As it can be seen in the segmented and labeled axial slice, Figure 8, the segmentation also allows to identify and label some of the main white matter structures, Corpus Callosum (CC), Anterior Corona Radiata (ACR), Forceps Major (fmajor) and Forceps Minor (fminor).

**Figure 9:**
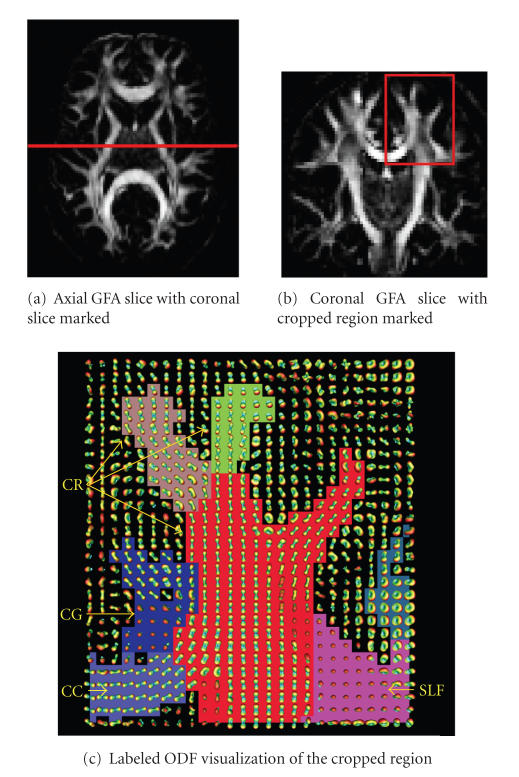
Our proposed algorithm is able to identify important white matter fiber bundles on a coronal slice of a real dataset. Generalized fractional anisotropy axial, Figure 9(a), and coronal, Figure 9(b) slices are shown. Labeled ODF visualization, Figure 9(c), each color represents one of the 7 clusters found. The white matter labels are CC: Corpus Callosum, CG: Cingulum, CR: Corona Radiata, SLF: Superior Longitudinal Fasciculus.

In Figure 9, the location of the cropped coronal slice is shown in the axial slice, Figure 9(a), and coronal slice, Figure 9(b). As it can be seen in the segmented and labeled coronal slice, Figure 9(c), the segmentation allows to identify and label main white matter structures: Corpus Callosum (CC), Cingulum (CG), Corona Radiata (CR), Superior Longitudinal Fasciculus (SLF). Note that the segmentation is resilient to crossing areas such as seen at the interface between CR and CC.

## 4. DISCUSSION

We have presented an algorithm to perform Q-ball imaging segmentation of white matter fiber bundles. The proposed method combines state-of-the-art HARDI reconstruction and state-of-the-art spectral clustering techniques. Our algorithm is initialization-free and has only two parameters. A scale-space parameter and the number of regions (clusters) are to be found. Regarding this number of clusters parameter, we have proposed to estimate it automatically. We have introduced a spectral embedding technique that does not require uniform sampling of the elements. To do so, the affinity measure used incorporates an Euclidean distance measure between the spherical harmonic coefficients describing the Q-ball ODFs and also incorporates the spatial location distance between ODFs. The affinity measure and the metric induced in the embedded space is then used to cluster Q-ball ODF images into multilabel segmentation representing the fiber bundles. Spectral embedding has already been applied to dMRI (e.g., [5]). However, to our knowledge, this is the first work using the diffusion maps that avoids the high dependence on element sampling. It is also the first work attempting Q-ball ODFs.

We have illustrated that the ODFs are the desirable elements to use for clustering in the white matter because the classical DT model is limited in regions of fiber crossings. The ODF is even more attractive because of the recent analytical spherical harmonic solution to the ODF reconstruction [3, 8, 9, 13]. The analytical solution is in fact as fast as a standard DT least-square estimation. In this work, we believe that we have used the state-of-the-art ODF reconstruction method [13], which is regularized, robust and very simple to implement.

The spectral embedding performed by the diffusion maps technique is at the heart of our segmentation algorithm. Whereas other spectral embedding techniques have a tendency to produce artifacts in the presence of different sampling characteristics within a cluster, the technique used in this work greatly reduces this tendency by performing the simple linear algebra calculation shown in (17).

Spectral embedding techniques produce a representation of the embedded data based on element-to-element affinities. This leads to the fundamental issue: how to choose the affinity measure? It is a challenge to find a measure that incorporates similarities between elements as well as the spatial location difference between elements. For similarities between elements, we chose the Euclidean distance between spherical harmonic coefficients describing the ODFs. This approach is simple and very efficient because it allows to process the ODFs directly on the SH coefficients. The Euclidean distance has also been used successfully in a level set segmentation framework [12] and it would be interesting to compare our spectral clustering approach against it. For spatial location difference, we chose Markovian relaxation in order to be consistent with the graph theoretical representation of the diffusion maps technique. Although this way of representing the distance involves an artificial elimination of all the nonneighboring relations of the ODF elements in the affinity matrix and an adjustment of the diagonal elements, we believe that the resulting affinity relations represent the affinity better. The affinity of two neighboring elements at the beginning of the Markovian relaxation algorithm is represented by a function of the Euclidean distance between them. This affinity can be interpreted as the probability that a random walker has of going from the first element to the second. The affinity of two elements at the end of the relaxation is the probability of a random walker starting from one element and reaching the second in a certain number of steps.

The final step of our algorithm is k-means clustering. We believe that there is room for improvement in this last part of the algorithm. In the first place, the k-means algorithm needs an explicit number of clusters to find. This can be heuristically determined by analyzing the eigenvalue plot or the reordered affinity matrix structure, as shown in this work. However, an automatic method that could find the number of clusters would considerably improve the algorithm. In the second place, the k-means algorithm and its variants, for instance, k-medians, k-medioids, search for isotropic clusters in the embedding space [25]. These methods are able to perform clustering on convex structures. This could also improve the last clustering phase of our algorithm

Finally, in order to analyze the importance of the difference between our diffusion maps algorithm and the widely used N-Cuts, we used synthetic simulations. In these simulations, we generated a synthetic image with a single cluster within which the sampling of the elements changed. We showed that when this sampling changes, the N-cuts algorithm produces artifacts while our diffusion maps method does not. As uniform sampling within a cluster is a difficult property to guarantee in the white matter fiber bundles, our diffusion maps method is better suited for this task. We thus believe that diffusion maps are the right clustering method to be used on dMRI processing problems.

## 5. CONCLUSIONS

In this work, we have presented two contributions. First, we have shown that in order to perform spectral clustering on complex dMRI with crossing fiber bundles, an HARDI technique such as Q-ball imaging is better than the classical DTI technique. This is because the ODF reconstructed from QBI is able to recover multiple crossing fiber populations. Second, a diffusion maps-based technique for image segmentation was introduced to reduce artifacts arising from the widely used N-Cuts image segmentation. We have illustrated the advantages of the ODF diffusion maps segmentation algorithm, and showed on a real dataset that our algorithm is able to identify important and complex white matter fiber bundles.

Finally, the diffusion maps technique has been shown to be more robust to sampling frequency variations within each object to be segmented. In order to cluster the elements, we have used an adaptive scale-space parameter and we have used Markovian relaxation in order to incorporate spatial dependencies. Overall, the approach is theoretically sound with the graph-based representation which lies at the heart of spectral clustering methods.

Therefore, we have an algorithm to perform fiber bundle clustering for a single brain. It is now important to study the behavior over several subjects in order to assess the reproducibility of the algorithm. In time, this will enable to perform multisubject statistics within bundles in the embedded space. This will help characterize the white matter fiber bundles of several subjects and study if the alteration of these segmented tracts can provide new biomarkers for white matter diseases.

## Footnotes

^1^ In this section, for simplicity, the subindexed variables $f_{i}$, $f_{j}$ represent different elements to be clustered and not spherical harmonic coefficients as in Section 2.2. $f_{i}$ is the full ODF element $\in {\mathbb{R}}^{R}$ at position i.
